# Structural and chemical biology of deacetylases for carbohydrates, proteins, small molecules and histones

**DOI:** 10.1038/s42003-018-0214-4

**Published:** 2018-12-05

**Authors:** Marco Bürger, Joanne Chory

**Affiliations:** 1 Plant Biology Laboratory, Salk Institute for Biological Studies, 10010 North Torrey Pines Road, La Jolla, CA 92037 USA; 2 Howard Hughes Medical Institute, Salk Institute for Biological Studies, 10010 North Torrey Pines Road, La Jolla, CA 92037 USA

**Keywords:** Pathogens, Carbohydrates, Acetylation, Histone post-translational modifications, Enzyme mechanisms

## Abstract

Deacetylation is the removal of an acetyl group and occurs on a plethora of targets and for a wide range of biological reasons. Several pathogens deacetylate their surface carbohydrates to evade immune response or to support biofilm formation. Furthermore, dynamic acetylation/deacetylation cycles govern processes from chromatin remodeling to posttranslational modifications that compete with phosphorylation. Acetylation usually occurs on nitrogen and oxygen atoms and are referred to as N- and O-acetylation, respectively. This review discusses the structural prerequisites that enzymes must have to catalyze the deacetylation reaction, and how they adapted by formation of specific substrate and metal binding sites.

## Abstract (web summary)

Marco Bürger and Joanne Chory discuss the structural requirements for enzymes carrying out deacetylation reactions for various functions across phyla. They explore how these enzymes have adapted to and achieved specificity on a large number of target molecules.

## Introduction

Intuitively, a deacetylation reaction requires prior acetylation. The introduction of an acetyl group into a molecule results in an amide bond formation upon nitrogen acetylation (N-acetylation) and in the creation of an ester bond if an oxygen is being acetylated (O-acetylation). Historically, N-acetylation of biomolecules has received overwhelming attention over O-acetylation, due to the discovery of outstanding and very general regulatory biological features: In 1976, co-translational N-terminal protein acetylation was first reported^1^, a modification that has a profound impact on protein stability and localization. The reaction is catalyzed by N-terminal acetyltransferases (NATs), six of which have been identified in humans so far. Another important discovery was the acetylation/deacetylation dynamics that control histone function run by histone acetyltransferase (HAT) and histone deacetylase (HDAC) activities^2^, and the fact that acetylation is required to activate the tumor-suppressor protein p53^3^. Another prominent example is the acetylation of lysine 40 on α-tubulin, which is a requirement for stable microtubule formation^4–6^. Compared to these important targets, O-acetylation has remained much less explored. The most prominent example has been the acetylation of serine and threonine residues by the bacterial YopJ effector family. The plague bacterium *Yersinia pestis* uses acetyltransferases to acetylate phosphosites within the MAPK pathway, thereby cutting off signal transduction in the host cell and sabotaging immune response^7^. The YopJ superfamily of acetyltransferases is conserved in animal and plant pathogenic bacteria^8^, and as a matter of fact, the structural details about YopJ activation and mechanism are known from studies about HopZ1a and PopP2, which are produced by the phytopathogens *Pseudomonas syringae* and *Ralstonia solanacearum*, respectively^9,10^. It remains unclear whether protein O-acetylation is exclusively a strategy used by pathogens or whether it is also a common, native reaction, which dynamically regulates the cellular phosphoproteome.

**Fig. 1:**
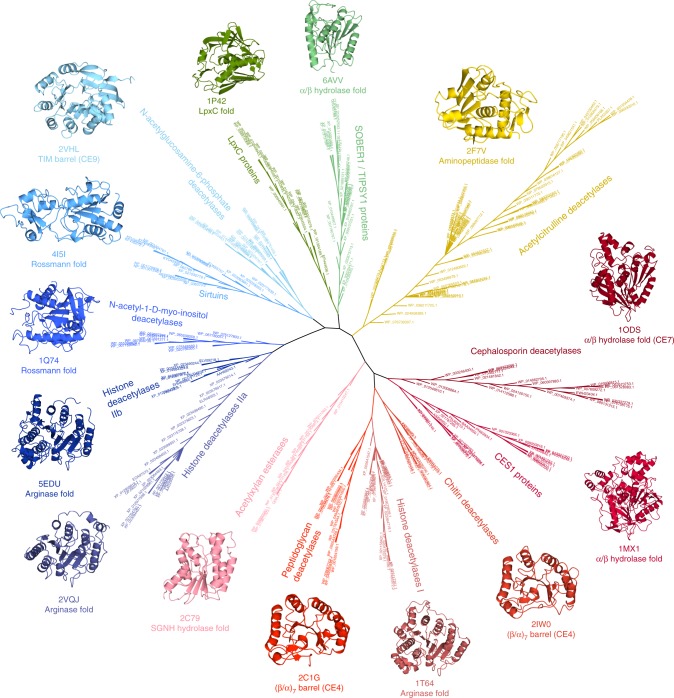
Phylogenetic tree of the deacetylase families discussed in this review, showing NCBI reference sequences (RefSeq)^87^ accession numbers. Created with SeaView 4.5.4^88^. All protein structures herein were visualized with CCP4mg^89^. Proteins discussed in this review were taken to represent each family and are displayed showing their folds, PDB codes and annotations from the CAZy database^90^ (CE4, CE7, and CE9)

**Fig. 2:**
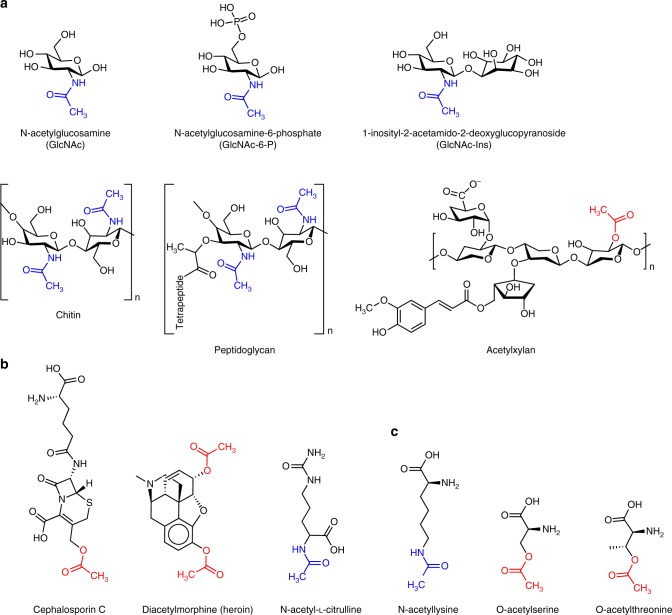
Overview of the diversity of deacetylase substrates discussed in this review. **a** Carbohydrates, **b** small molecules, and **c** amino acid residues. N-acetyl are highlighted in blue and O-acetyl in red, respectively. Chemical structures were drawn using ChemDraw (Perkin Elmer)

Besides the acetylation and deacetylation of amino acids, we discuss how the high number of acetylated targets and different target molecules has led to the evolution of an evenly high diversity of deacetylating enzymes (Fig. 1). These—while having basic catalytic chemistry in common—are characterized by specific structural features that allows them to recognize a high variety of substrates: For example, the majority of acetylated carbohydrates such as chitin, peptidoglycan, and N-acetylglucosamine are modified with acetyl groups via an amide bond on their nitrogen atoms^11^, with the exception of acetylxylan, which is O-acetylated via an ester linkage (Fig. 2a). Finally, we outline how small metabolic molecules with O-linked acetyl groups such as the antibiotic cephalosporin C or the opioid heroin (Fig. 2b) usually get deacetylated by promiscuous esterases^12,13^ and discuss the role of deacetylases in plant immunity.

## Carbohydrate de-N-acetylases

**Fig. 3:**
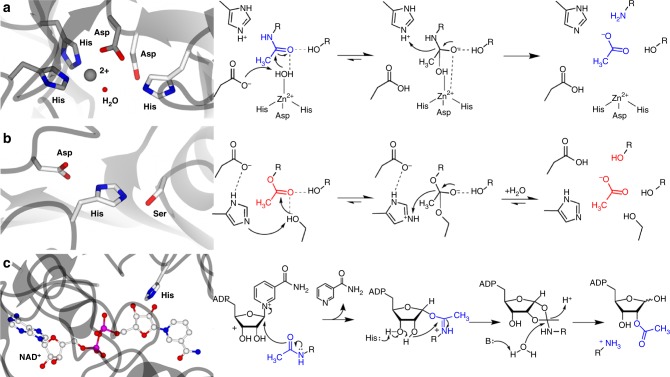
Deacetylases can be classified in three major groups, according to their catalytic sites. Exemplary active site arrangements and simple catalytic mechanisms of **a** de-N-acetylases (PDB code 4F9D^25^), **b** de-O-acetylases (PDB code 3M81^54^), and **c** class III HDACs (PDB code 4I5I^65^). N-acetyl is highlighted in blue and O-acetyl in red, respectively. Typical catalytic site residues are shown with white carbon atoms and metal binding residues in **a** are shown with carbon atoms colored in gray

**Fig. 4:**
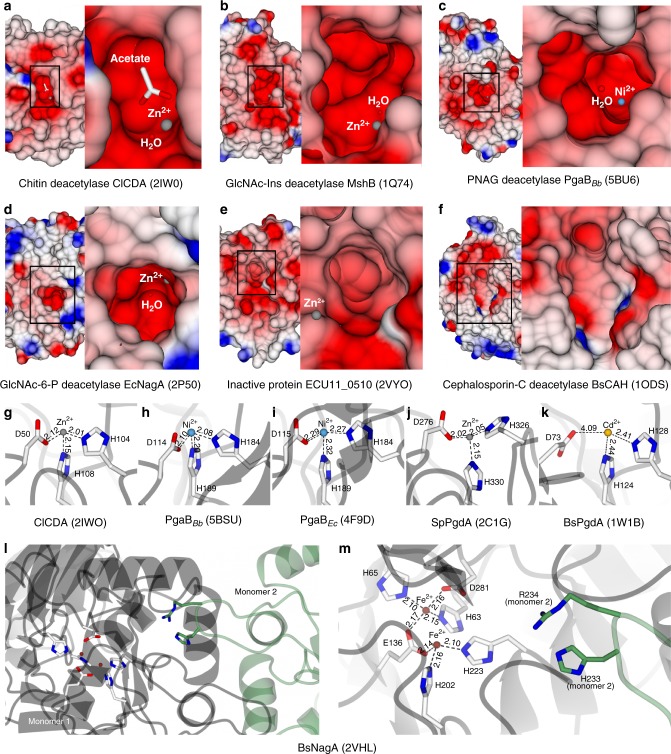
Deacetylases feature a signature binding groove with electronegative charge. **a**–**f** Protein surface representations of deacetylases with different substrate specificities. Electrostatic surface potentials are contoured from −12.8 kT e^−1^ (red) to +12.8 kT e^−1^ (blue). **g**–**k** Divalent metal coordination by different deacetylases. Distances are in Å. **l** Dimeric composition and **m** metal binding sites (white) and substrate binding residues (green) of the N-acetylglucosamine-6-phosphate deacetylase from *Bacillus subtilis*

Oligomerized sugars are among the most abundant and most durable building blocks in life^14^. Their diversity ranges from chitin, which makes up the exoskeleton of insects and the cell walls of fungi to peptidoglycan, the constitutive cell wall component of many bacteria. Remodeling of these oligomers by deacetylation has proven crucial for pathogenesis and host immune evasion. Most of these modifications are N-acetylation events and therefore, despite different folds, one common feature of the deacetylating enzymes can be found in their active sites. To break the amide bond, a His–His–Asp triad that binds a divalent metal cation and a catalytic Asp–His pair with a nearby water molecule are an often-found architecture^15,16^. The catalytic base aspartate will abstract a proton from the water, making it a nucleophile. This deprotonation is aided by the metal ion, which decreases the p*K*_a_ of the water. The catalytic acid histidine protonates the nitrogen-bound reaction intermediate, breaking the amide bond and leaving a free amine and an acetate^17,18^. This mechanism holds true not only for acetylated sugar oligomers but for N-acetylation in general (Fig. 3a). Thus, the main differences between the enzymes lie in their surface topology, accommodating substrates of different shapes and sizes. The crystal structure of the chitin deacetylase ClCDA from the fungal plant pathogen *Colletotrichum lindemuthianum* revealed a (β/α)_(7)_ barrel fold and a prominent cleft with strong negative charge as binding site for the acetyl group^19^ (Fig. 4a). Deacetylated chitin (chitosan) is a very poor substrate for chitinases^20^; therefore deacetylation helps avoiding the creation of chitin breakdown products, which would otherwise be recognized by the plant’s immune system.

The crystal structure of 1-d-myo-inosityl 2-acetamido-2-deoxy-alpha-d-glucopyranoside deacetylase (MshB) from *Mycobacterium tuberculosis* revealed a typical Rossman fold but also features a strong electronegative surface cleft, which has a deeper depression compared to the chitin deacetylase (Fig. 4b)^21^. The substrate GlcNAc-Ins is not as large as the polymeric chitin; therefore, a more profiled binding cleft has evolved, providing a site located deeper in the protein to bind the acetyl group. GlcNAc-Ins is a precursor in the biosynthesis of mycothiol^22^, an important reducing agent in actinomycetes including mycobacteria, helping to control reactive oxygen species. In contrast, the periplasmatic, metal-dependent poly-acetyl-d-glucosamine (PNAG) deacetylase from *Bordetella bronchiseptica* (BpsB) displays a (β/α)_(7)_ barrel fold and a surface topology characterized by a more distinct electronegative spot for binding of the acetate, surrounded by less negative charge^23^. Deacetylation of PNAGs is crucial for *Bordetella* biofilm stability and colonialization of the respiratory tract. A recent study about PgaB and BpsB has revealed that the proteins’ C-terminal domains, which were previously thought to be catalytically inactive, have glycoside hydrolase activity^24^. This activity requires deacetylated PNAGs. The current model, therefore, suggests a sequential degradation of PNAG molecules by PgaB proteins, first by deacetylating the substrate and then by hydrolyzing the glycosidic bonds. For reasons of clarity, the authors of the above-mentioned publication have also introduced new names for *B. bronchiseptica* BpsB and *E. coli* PgaB, which are now referred to as PgaB_*Bb*_ and PgaB_*Ec*_, respectively. In the crystal structure of PgaB_*Bb*_, a nickel ion was found in the catalytic center. Biochemical tests showed the highest enzyme activity with Ni^2+^ and Co^2+^, and the *Escherichia coli* homolog PgaB_*Ec*_ was found with a nickel cation in the metal binding site, too^25^. Compared to the chitin deacetylase ClCDA, the substrate binding site of PgaB_*Bb*_ is deeper, and the metal ion is located more towards the center of the cavity (Fig. 4c). The distances of the metal ion to the side chains of the His/His/Asp triad are smaller in the zinc-bound structure of ClCDA than in both nickel-bound structures of PgaB_*Bb*_ and PgaB_*Ec*_ (Fig. 4g–i). The higher mass of the atomic nucleus of zinc compared to nickel causes a more contracted electron shell and consequently a smaller atomic van der Waals radius of the zinc atom. The atomic van der Waals radii of zinc and nickel are 1.39 and 1.63 Å, respectively, and generally, the van der Waals radii decrease with an increasing atomic mass within elements 19–30 in period 4 of the periodic table^26^. Therefore, a slightly larger metal binding site might be needed to accommodate a cobalt or nickel compared to zinc. Although the differences in these distances are rather subtle, they might be the reason why cobalt and nickel increase biochemical activity of PgaB while the smaller zinc does not. A smaller metal might be bound to the metal-binding triad with insufficient affinity or might be inefficiently positioned towards the catalytic water.

Compared to both PgaB_*Bb*_ and PgaB_*Ec*_, the crystal structure of the N-acetylglucosamine-6-phosphate (GlcNAc-6-P) deacetylase from *E. coli* folds into a TIM barrel architecture and the protein surface features a much less electronegative charge around the substrate binding site^27^. Instead, the protein surface is more hydrophobic around a distinct cavity of sharp electronegative charge (Fig. 4d). Next to the entrance to the site, an electropositive spot for the coordination of the phosphate group of the GlcNAc-6-P molecule is located. The catalytic centers of several solved structures of GlcNAc-6-P deacetylases have turned out to be relatively different regarding the side chains involved in metal and substrate binding: The above-mentioned enzyme from *E. coli* features a His/His/Glu metal binding site and a catalytic center composed of Asp/Asn/Gln plus the nucleophilic water^28^. However, these asparagine and glutamine residues are substituted by two histidines in the homologous enzyme from *Thermotoga maritima* (PDB code 1O12). The ortholog from *Bacillus subtilis* incorporates two divalent iron ions. It is assumed that one of the two metals facilitates the nucleophilic attack activated by the other metal through stabilization of the substrate^29^. Additionally, the active GlcNAc-6-P deacetylase in *B. subtilis* is a dimer (Fig. 4l), in which the His/His/Glu and His/His/Asp metal binding sites are being contributed by one monomer and the substrate binding residues by the other monomer. Only upon dimerization do the two parts come close enough to form an active substrate binding site (Fig. 4m).

Another protein family, peptidoglycan deacetylases, also facilitate bacterial evasion of host immune response and are, therefore, important virulence factors. In particular, deacetylated peptidoglycan is not recognized by mammalian lysozyme, which requires the N-acetyl group for catalysis^30^. The peptidoglycan GlcNAc deacetylase from *Streptococcus pneumoniae* displays a prominent electronegative cleft on the protein surface, constituting the substrate binding groove^18^. The cleft is surrounded by charge as negative except for a few electropositive spots on the side. The homolog from *B. subtilis* possesses a more hydrophobic surrounding but both proteins have a His/His/Asp metal binding site and a His/Asp/water catalytic center in common^31^. Both proteins have a (β/α)_(7)_ barrel structure. Interestingly, the *Streptococcus* enzyme was found with a zinc ion coordinated inside the substrate binding site while attempts to bind zinc to the *Bacillus* homolog failed. Instead, it was eventually complexed with cadmium. A comparison of the two metal binding sites showed longer distances between metal-coordinating residues in the *B. subtilis* structure compared to *S. pneumoniae* (Fig. 4 j–k), therefore a larger ion might be needed for activity (cadmium has a van der Waals radius of 1.58 Å compared to 1.39 Å of zinc). In 2009, the crystal structure of a biochemically uncharacterized, putative amidase from the eukaryotic parasite *Encephalitozoon cuniculi* was published^32^. No activity on any tested substrate has been observed and distortions in the protein’s active site arrangements underlined the importance of the correct positioning of the parts that compose substrate binding site and catalytic center. The shape of the substrate binding groove looks dissimilar to the ones found in the previously discussed enzymes, and the active site metal ion is located more than 5 Å away from the position found in other deacetylases (Fig. 4e). In addition, the reaction-activating aspartic acid has shifted away from the active site. Therefore, the enzyme appears either generally catalytically inactive or it binds a molecule structurally much different than previously tested sugar substrates.

Another important enzyme implicated in pathogenicity is UDP-(3-O-(R-3-hydroxymyristoyl))-N-acetylglucosamine deacetylase (LpxC). It catalyzes the first committed step of lipid A biosynthesis^33,34^. Lipid A is the membrane anchor of lipopolysaccharide (LPS) or endotoxin, the major component of the outer membrane of Gram-negative bacteria^35^. LpxC has a unique overall fold that does not match any other known architectures. LpxC has a zinc binding site composed of an aspartate and two histidines^36^. The surface of the protein features a hydrophobic tunnel going through the upper part of the protein to accommodate the lipid moiety and a strong electronegative substrate binding site. It had been unclear whether a bifunctional general acid–base glutamate in the active site would promote the nucleophilic attack of a water, with a histidine stabilizing the oxyanion intermediate^37^, but without acting as a general acid or if the glutamate and histidine act as a typical general acid–base catalyst pair^38^. The first mechanism is similar to the one found for carboxypeptidase A^39^. Here, the glutamate activates the nucleophilic zinc–water complex, but it also facilitates the collapse of the tetrahedral reaction intermediate through partial protonation of the amine leaving group. In the second mechanism, the glutamate also activates the nucleophilic zinc–water complex, but the amine leaving group is protonated by the histidine, leading to the breakdown of the tetrahedral intermediate. More recent crystal structures have supported the latter model^40^.

## Carbohydrate de-O-acetylases / acetylxylan esterases

Acetylxylan esterases or acetylxylan deacetylases are part of a concerted enzymatic action to break down plant cell wall xylan^41–43^, and they usually target the O-2 and/or O-3 position on the acetylxylan molecule^44^. A 2006 study revealed the crystal structures of two related metal-dependent xylan esterases from *Streptomyces lividans* and *Clostridium thermocellum*^45^. The *S. lividans* protein turned out reminiscent of the structural organization known from sugar de-N-acetylases, with a (β/α)_(7)_ barrel fold and a His/His/Asp triad binding a zinc cation. In contrast, the *C. thermocellum* homolog displays a different loop conformation in the metal binding center plus a histidine to tyrosine substitution, leading to a cobalt ion being coordinated by an aspartate and a histidine. The second histidine is found replaced by four water molecules. In both structures the nucleophilic water is accompanied by a catalytic base aspartate and a catalytic acid histidine. Interestingly, both enzymes displayed the highest activity with Co^2+^, however, while the *S. lividans* protein retained a third of its activity when provided Zn^2+^, the activity of the *C. thermocellum* homolog was almost completely diminished. Both enzymes were able to tolerate manganese.

Other xylan esterases have a different architecture: The structure of acetylxylan esterase (AXE II) from the fungus *Penicillium purpurogenum* revealed a canonical SGNH hydrolase fold with a catalytic triad comprising a nucleophilic serine, a catalytic acid histidine and a catalytic base aspartate^46^. The mechanism of de-O-acetylation is somewhat similar to de-N-acetylation but does not involve a nucleophilic water or a metal ion. The reason for this is the presence of a serine residue. Upon substrate binding, the aspartate in the catalytic triad forms a low-energy barrier with the histidine, increasing the histidine’s p*K*_a_ on the imidazole ring. The histidine will act as a general base and abstracts a proton from the hydroxyl group on the serine, making it nucleophilic. A tetrahedral reaction intermediate is then formed. The proton is transferred to the ester bond, which breaks upon protonation (Fig. 3b). A later crystal structure of AXE II from the thermophilic soil bacterium *Geobacillus stearothermophilus* showed the same organization of the catalytic site as the *P. purpurogenum* enzyme. Interestingly, the *G. stearothermophilus* protein turned out to be an octamer, which was confirmed in solution^47^. A possible reason for this quaternary structure could be either specificity or higher catalytic efficiency since this organization moves the active sites of the monomers in close proximity to each other. The hydrogen bonds and *π*-stacking interactions that stabilize the octamerization are located closely to the active sites, suggesting that the multimeric arrangement might stabilize the catalytic loops. Indeed, a follow-up study has mentioned dimerizing mutations of the protein that lead to a reduction in enzymatic activity^48,49^. Alternatively, the assembly might simply increase stability, considering that *G. stearothermophilus* is a thermophilic organism.

## Multimeric cephalosporin-C and multi-substrate esterases

**Fig. 5:**
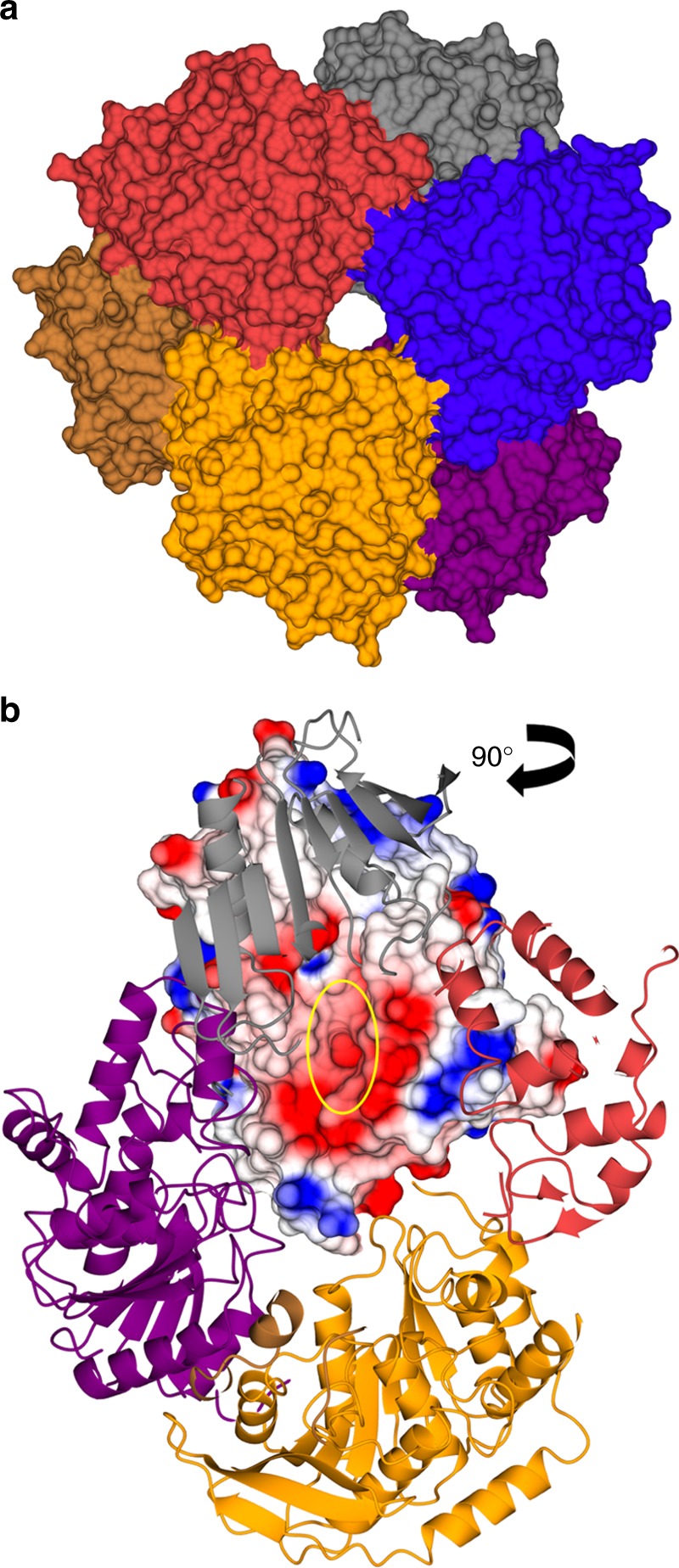
Some deacetylases achieve specificity through oligomerization. **a** Oligomerization properties of small molecule deacetylases highlighting the narrow tunnel entrance to the hexamer and **b** the active sites (yellow circle) positioned towards the inside of the substrate conduit (PDB code 1ODS^12^). The electrostatic surface potential is contoured from −12.8 kT e^−1^ (red) to +12.8 kT e^−1^ (blue)

Cephalosporin C^50^ was first isolated from the fungus *Acremonium*^51^ and —like most other β-lactam antibiotics such as penicillin^52^—targets bacterial cell wall biosynthesis^53^. The acetyl group in cephalosporin C makes it a target for bacterial deacetylases, the activity of which leads to inactivation of the chemical. The structure of a cephalosporin C esterase from *Thermotoga maritima* has been solved and revealed a classical α/β hydrolase fold and a catalytic Ser/His/Asp triad. The protein features an electronegative binding site for the acetyl group right after the active site serine and an additional, larger cavity immediately adjacent to it^54^. This second depression most likely serves to accommodate the bulky core structure of the cephalosporin molecule. A homologous structure from *Bacillus subtilis* showed an almost identical fold but displayed a less electronegative charge on the surface and a smaller binding groove (Fig. 4f), possibly indicating a slightly divergent substrate specificity^12^. One additional and interesting feature is the higher oligomeric order of the proteins. The enzymes are native hexamers, with their active sites pointing inwards a substrate tunnel, making their assemblies reminiscent of the self-organizing proteasome architecture (Fig. 5). In one of the *T. maritima* structures, the entrance to this tunnel is covered by several phenylalanine residues, indicating a possible gatekeeper role for these amino acids and a selection towards more hydrophobic molecules. Both surface topology and multimerization appear to contribute to the substrate specificity of these enzymes, which is not limited to cephalosporin C but it is likely that they serve as a general de-O-acetylation conduit for small molecules.

Another example of a hexameric and promiscuous detoxification deacetylase is human carboxylesterase 1 (CES1)^13^. CES1 also folds into a canonical α/β hydrolase architecture with a classical Ser/His/Asp catalytic triad^55^. The specificity towards small molecules in likely given by its oligomerization properties, with the enzyme known for deacetylation of cocaine and heroin. CES1 exists in a trimer-hexamer equilibrium, with the hexamer composed of two stacked trimers. Binding of a small molecule to the allosteric Z-sites of the hexamer causes it to separate into two active trimers. Like the active site, the allosteric site is promiscuous too, and only the size of the molecule appears important for binding to the Z-site. The reason for this allosteric regulation is not fully understood but the fact that the inside of the trimer is lined up with hydrophobic side chains (except for the active sites) might require a higher oligomeric organization for protein stability.

## Histone / lysine deacetylases

Modification of histones has an important role in regulation of chromatin structure and gene activity^56^, and are modulated by the activities of histone acetyltransferases (HATs) and histone deacetylases (HDACs)^2^. The acetylation occurs on lysine residues lying in the N-termini of core histones^57^ but the activity of HDACs is not limited to histones. HDAC6, for instance, deacetylates α-tubulin^58^. The enzymes’ cofactors divide them into two major groups: zinc and NAD^+^ dependent^59,60^. Whereas zinc-dependent HDACs are characterized by a canonical arginase fold, the NAD^+^-dependent class displays a typical Rossmann architecture (Table 1, Fig. 1), a structural motif frequently found in proteins that bind nucleotides. Sequence homology classifies them in class I, IIa, IIb, and III HDCAs. Class I enzymes are expressed in all tissues in humans and mice, and they feature a zinc binding site made of an Asp/Asp/His triad and a catalytic site composed of two histidines, one tyrosine, and a nucleophilic water. They also bind a potassium ion about 7 Å away from the catalytic zinc that might have a role in substrate binding^61^. Besides their expression in a tissue-specific manner, one feature that distinguishes class IIa HDACs from class I enzymes is the presence of an additional non-catalytic but structural zinc ion. This additional metal is coordinated by two histidines and two cysteines, which are strictly conserved within the class IIa family but absent in other HDACs^62^.

Class IIa HDACs are catalytically weak deacetylases, due to the substitution of the catalytically important tyrosine by a histidine, and histones are not their substrates. The current thinking, therefore, has accepted a non-catalytic role for class IIa HDACs. Only their interaction in a complex with SMRT/N-CoR and HDAC3, a class I HDAC, provides catalytic activity, which entirely comes from the associated HDAC3 protein^63^. Class IIb members strongly resemble class I enzymes, with slight differences in how the catalytic machinery functions. A study of the class IIb HDAC6 suggests that the tandem histidines in the catalytic site occupy separate roles as general base and general acid^64^. In HDAC8, a class I HDAC, the second histidine serves as both, general base and acid^61^.

Class III HDACs, also called sirtuins, have an NAD^+^-dependent mechanism. The NAD^+^ forms a ternary complex with the enzyme and the substrate. Nicotinamide is then released from the NAD^+^, followed by the transfer of the acetyl group from the substrate onto the ADP-ribose, leaving the substrate’s lysine deacetylated. Thus, sirtuins possess both deacetylase and ADP ribosyl transferase activities^65^. All sirtuins seem to have a conserved histidine residue that acts as the general base, deprotonating one of the ribose oxygens^66^ (Fig. 3c).

In addition to these groups, plants express another family of HDACs, called type-2 HDACs (HD2s). HD2s were first identified in maize^67^, and have since been found in almost all land plants. About 60% of HD2s contain a zinc-finger domain and HD2s without this domain are only found in angiosperms^68,69^. HD2s seem to be involved in leaf development^70^ and abiotic stress response^71^, both of which are plant-specific functions. Although there are reports suggesting that HD2s might have deacetylase activity, as they seem to have a role in the deacetylation of nuclear proteins^72^, and are able to modulate levels of histone acetylation^73^, direct biochemical or structural evidence proving that they are deacetylases is still missing.

## N-acetyl-l-citrulline deacetylases

The first step in arginine biosynthesis is the acetylation of glutamate. This is followed by the so-called acetyl cycle, in which the acetyl group gets passed on from glutamate, finally leading to the formation of N-acetyl-l-ornithine. N-acetyl-l-ornithine will then be deacetylated, recycling the acetyl group onto glutamate, thereby regenerating N-acetyl-l-glutamate and yielding l-ornithine, an arginine precursor^74^. This last step is catalyzed by the enzyme acetylornithine deacetylase^75^. However, arginine biosynthesis in Proteobacteria follows a different path and does not involve the direct deacetylation of l-ornithine but rather a transcarbamoylation into N-acetyl-l-citrulline and its deacetylation to l-citrulline^76,77^. Thus, a different enzyme is needed: N-acetyl-l-citrulline deacetylase (ACDase). In 2007, the crystal structure of ACDase from *Xanthomonas campestris* was solved^78^. The protein possesses a metal binding site of His/Asp/Glu that coordinates a cobalt ion within an aminopeptidase architecture. The cobalt is in contact with the nucleophilic water within the catalytic site comprising a glutamate as a single bifunctional general acid–base catalyst. The binding site constitutes a deep electronegative cleft, with a long hydrophobic arm forming the C-terminal domain. The fact that ACDases are Proteobacteria-specific, which involves major pathogens, makes it an interesting drug target.

## Plant protein deacetylases

Recently, a family of plant-specific protein deacetylases has been identified that are implicated in suppression of the host’s hypersensitive response (HR) against pathogens. The protein is called SOBER1, an α/β hydrolase highly related to acyl-protein thioesterases^79^. An insertion in the catalytic lid-loop renders the protein’s specificity into a protein deacetylase by blocking the hydrophobic tunnel that would otherwise accommodate longer substrates. The SOBER1 family can be further broken down into a TIPSY1 group, which possesses deacetylase activity but does not suppress hypersensitive response^80^. The proteins are highly promiscuous and SOBER1’s confirmed deacetylation substrates so far are the bacterial effector protein AvrBsT and the microtubule-associated ACIP1, which in turn is a substrate of AvrBsT. Another recent story reported that SOBER1 is able to suppress HR elicited by multiple bacterial acetyltransferases^81^. Though specific deacetylation sites are not known to date, the lack of a metal ion and the presence of a classical Ser/His/Asp catalytic triad suggest that SOBER1 and TIPSY1 are protein de-O-acetylases and are unable to break amide bonds.

## Potential human protein de-O-acetylases

Reports about human protein deacetylases have exclusively focused on histone/lysine deacetylases and until today, no protein de-O-acetylases have been identified. The above-mentioned SOBER1/TIPSY1 family is only conserved in plants and the oligomerization properties of the promiscuous human CES1 esterase likely limits its substrate scope to small molecules. However, a protein called LYPLAL1 has been identified as an enzyme with disputed catalytic function. Genetic association studies have suggested its role in fatty acid metabolism and the protein acting as a phospholipase^82–84^. Like SOBER1, it is structurally related to acyl-protein thioesterases (APTs) and it was long thought to be the third human APT^85^. However, a structural study concluded that a different loop conformation impairs the substrate binding tunnel, changing LYPLAL1’s substrate specificity towards short acyl groups^86^. The presence of a Ser/His/Asp catalytic triad and the fact that LYPLAL1 is a monomer suggest a possible function as a protein de-O-acetylase.

## Summary and outlook

Deacetylation events occur on a high number of chemically very different biomolecules (Fig. 2). Some reactions manipulate cell surface features to evade host immune response, others are involved in the regulation of basic cellular events and or counter-react prior acetylation events by bacterial effector proteins. Based on their catalyzed reaction, deacetylases can be divided into major groups: Metal-dependent de-N-acetylases and de-O-acetylases featuring a classical catalytic triad (Table 1). All deacetylases possess a distinct binding site for the acetyl group on their protein surface, characterized by a strong electronegative charge. Thus, substrate specificity originates from the immediate surrounding of the acetyl binding pocket. Further specificity appears to be given by oligomerization properties, creating a tunnel-like structure with a molecular weight cutoff selecting for small molecules.

**Table 1:** Overview of the proteins and their molecular features discussed in this review

Name	PDB code	Substrate	Co-factor	Catalytic domain	Biological function
**Carbohydrate deacetylases**					
*Colletotrichum lindemuthianum* CDA	2IW0	Chitin	Zn^2+^	(β/α)_(7)_ barrel	Fungal chitin deacetylation, evasion of plant host response
*Mycobacterium tuberculosis* MshB	1Q74	GlcNAc-Ins	Zn^2+^	Rossmann fold	Biosynthesis of the bacterial reducing agent mycothiol
*Bordetella bronchiseptica* BpsB	5BU6	PNAG oligomers	Ni^2+^	(β/α)_(7)_ barrel	Biofilm formation
*Escherichia coli* PgaB	4F9D	PNAG oligomers	Ni^2+^	(β/α)_(7)_ barrel	Biofilm formation
*Escherichia coli* NagA	1YRR, 2P50	GlcNAc-6-P	Zn^2+^	TIM barrel	Murein recycling
*Thermotoga maritima* NagA	1O12	GlcNAc-6-P	Zn^2+^	TIM barrel	Murein recycling
*Bacillus subtilis* NagA	2VHL	GlcNAc-6-P	2 Zn^2^	TIM barrel	Murein recycling
*Streptococcus pneumoniae* PgdA	2C1G	Peptidoglycan	Zn^2+^	(β/α)_(7)_ barrel	Bacterial peptidoglycan deacetylation, evasion of immune response
*Bacillus subtilis* PgdA	1W1B	Peptidoglycan	Cd^2+^	(β/α)_(7)_ barrel	Bacterial peptidoglycan deacetylation, evasion of immune response
*Encephalitozoon cuniculi* U11_0510	2VYO	Unknown	Zn^2+^	(β/α)_(7)_ barrel	Unknown, likely inactive protein
*Aquifex aeolicus* LpxC	1P42	UDP-N-acetylglucosamine	Zn^2+^	LpxC fold	Lipid A biosynthesis
*Streptomyces lividans* Axe	2CC0	Acetylxylan	Zn^2+^	(β/α)_(7)_ barrel	Plant cell wall degradation
*Clostridium thermocellum* Axe	2C79	Acetylxylan	Co^2+^	(β/α)_(7)_ barrel	Plant cell wall degradation
*Penicillium purpurogenum* Axe2	1BS9	Acetylxylan		SGNH hydrolase fold	Plant cell wall degradation
*Geobacillus stearothermophilus* Axe2	3W7V	Acetylxylan		SGNH hydrolase fold	Plant cell wall degradation
**Small molecule deacetylases**					
*Thermotoga maritima* AxeA	1VLQ, 3M81	Cephalosporin C		α/β hydrolase fold	Xylooligosaccharide/Cephalosporin C hydrolysis
*Bacillus subtilis* CAH	1ODS	Cephalosporin C		α/β hydrolase fold	Xylooligosaccharide/Cephalosporin C hydrolysis
*Homo sapiens* CES1	1MX1, 1MX5	Small molecules		α/β hydrolase fold	Promiscuous multi-drug degradation
*Xanthomonas campestris* ACDase	2F7V	N-acetyl-l-citrulline	Co^2+^	Aminopeptidase fold	Arginine biosynthesis
**Histone deacetylases**					
*Homo sapiens* HDAC8	1T64	Acetyllysines on histones	Zn^2+^	Arginase fold	Histone deacetylation
*Homo sapiens* HDAC4	2VQJ	Acetyllysines on histones	Zn^2+^	Arginase fold	Histone deacetylation
*Homo sapiens* HDAC6	5EDU	Acetyllysines on histones	Zn^2+^	Arginase fold	Histone deacetylation
*Homo sapiens* SIRT1	4I5I	Acetyllysines on histones	NAD^+^	Rossmann fold	Histone deacetylation
**Protein deacetylases**					
*Arabidopsis thaliana* SOBER1	6AVV	Acetylated proteins		α/β hydrolase fold	Hypersensitive response (HR) in plant immunity

Targeting deacetylases with small molecules might be a promising strategy to improve human health and aid agriculture. Especially bacterial and fungal deacetylases constitute an interesting drug target, since they are involved in pathogenicity or bacteria-specific metabolism, such as evasion of the mammalian immune response^20,30^, amino acid biosynthesis^76^ or biofilm formation^23^. In addition, inhibitors for the SOBER1 family of protein deacetylases might be a strategy to support the plant’s immune response against pathogenic *Pseudomonas* and *Xanthomonas*, based on the findings that SOBER1 suppresses hypersensitive response in *Brassicaceae*^79,80^.

While histone acetylation/deacetylation is well researched, and studies have been carried out to map acetylation sites cell-wide, these attempts have exclusively focused on lysine acetylation. Future studies are, therefore, likely to address O-acetylation and de-O-acetylation events in a global manner. It is still unknown as to how serine and threonine acetylation is used to compete with and regulate the phosphoproteome. A direct competition has only been shown in a handful of cases and each time, a pathogen was involved. In addition, no animal protein de-O-acetylase has been confirmed yet and their prediction from the protein sequence is rather difficult since minor changes to the fold or surface topology can result in a fundamentally altered substrate specificity. Therefore, more data obtained from mass spectrometry and structural studies will be necessary until acetylomic studies catch up with the amount of attention that other posttranslational modifications are receiving.
