# Publisher Correction: Structural and chemical biology of deacetylases for carbohydrates, proteins, small molecules and histones

**DOI:** 10.1038/s42003-018-0258-5

**Published:** 2019-01-03

**Authors:** Marco Bürger, Joanne Chory

**Affiliations:** 1 Plant Biology Laboratory, Salk Institute for Biological Studies, 10010 North Torrey Pines Road, La Jolla, CA 92037 USA; 2 Howard Hughes Medical Institute, Salk Institute for Biological Studies, 10010 North Torrey Pines Road, La Jolla, CA 92037 USA

**Keywords:** Pathogens, Carbohydrates, Acetylation, Histone post-translational modifications, Enzyme mechanisms

Correction to: *Communications Biology* 10.1038/s42003-018-0214-4; Published online 05 December 2018

In the original published version of the article, the protein name for the carbohydrate deacetylase in *Geobacillus stearothermophilus* was missing from Table 1. The protein name, Axe2 (corresponding to PDB code 3W7V), should have appeared immediately following the species name. In addition, a space was inadvertently omitted between *Xanthomonas campestris* and ACDase in Table 1. The errors have been corrected in the HTML and PDF versions.

